# *EGFR* uncommon alterations in advanced non-small cell lung cancer and structural insights into sensitivity to diverse tyrosine kinase inhibitors

**DOI:** 10.3389/fphar.2022.976731

**Published:** 2022-09-16

**Authors:** Haiyan Xu, Guangjian Yang, Runze Liu, Yaning Yang, Weihua Li, Junling Li, Xuezhi Hao, Puyuan Xing, Yan Wang

**Affiliations:** ^1^ Department of Comprehensive Oncology, National Cancer Center/National Clinical Research Center for Cancer/Cancer Hospital, Chinese Academy of Medical Sciences and Peking Union Medical College, Beijing, China; ^2^ Department of Respiratory Medicine, Shandong Cancer Hospital and Institute, Shandong First Medical University and Shandong Academy of Medical Sciences, Taian, Shandong, China; ^3^ Guangdong Provincial Key Laboratory of Advanced Drug Delivery Systems and Guangdong Provincial Engineering Center of Topical Precise Drug Delivery System, Guangdong Pharmaceutical University, Guangzhou, Guangdong, China; ^4^ Department of Medical Oncology, National Cancer Center/National Clinical Research Center for Cancer/Cancer Hospital, Chinese Academy of Medical Sciences and Peking Union Medical College, Beijing, China; ^5^ Department of Pathology, National Cancer Center/National Clinical Research Center for Cancer/Cancer Hospital, Chinese Academy of Medical Sciences and Peking Union Medical College, Beijing, China

**Keywords:** non-small cell lung cancer, epidermal growth factor receptor, uncommon alteration, targeted therapy, tyrosine kinase inhibitor

## Abstract

**Background:** Approximately 10% of patients with non-small cell lung cancer (NSCLC) harbor uncommon epidermal growth factor receptor (*EGFR*) alterations. This study aims to investigate the therapeutic responses and predict the binding activity of different tyrosine kinase inhibitors (TKIs) for *EGFR* uncommon alterations.

**Methods:** Between May 2014 and June 2021, clinical outcomes of NSCLC patients harboring *EGFR* uncommon alterations who received diverse treatment modalities: first-generation (1G) *EGFR*-TKI, second-generation (2G) *EGFR*-TKI afatinib, chemotherapy, and 1G TKI in combination with chemotherapy as the initial therapy were retrospectively analyzed, and structural analysis for the binding activity of major uncommon subtypes G719A, S768I, and L861Q to different TKIs were predicted.

**Results:** A total of 102 NSCLC patients harboring *EGFR* uncommon alterations with treatment and survival outcomes were included and analyzed. The majority of patients presented compound mutations (54.9%), and G719X plus S768I was the predominant subtype (*n* = 33, 32.3%). There was a significant difference in median progression-free survival (mPFS) between therapeutic patterns (*p* = 0.015) and *EGFR* alteration subtypes (*p* = 0.017). Rather than almonertinib and furmonertinib, afatinib, dacomitinib and osimertinib revealed favorable binding activity to G719A mutation. In contrast, S768I and L861Q mutation indicated an unaffected binding activity to these diverse kinds of *EGFR* TKIs.

**Conclusion:** Together with afatinib, 1G-TKIs combined with chemotherapy might be another effective option for NSCLC patients harboring *EGFR* uncommon alterations. Based on computational findings, afatinib, dacomitinib, and osimertinib might confer favorable activity to G719A, S768I, and L861Q, whereas almonertinib and furmonertinib revealed less activity to G719A.

## Introduction

**FIGURE 1:**
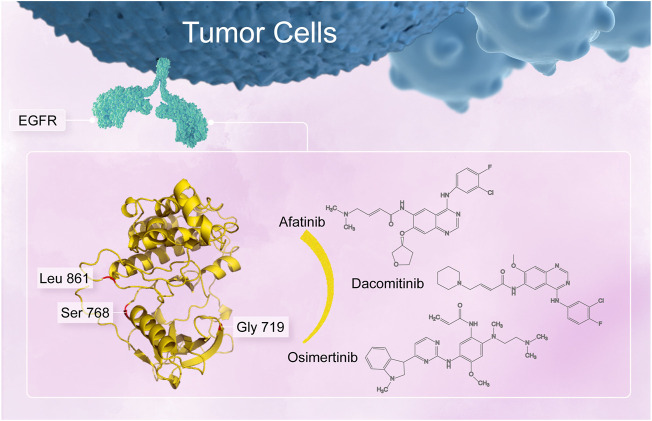
Uncommon alterations located in the amino acid residue G719, S768, and L861 in the EGFR tyrosine kinase domain.

Treatment paradigm for advanced non-small cell lung cancer (NSCLC) has dramatically improved over the past 2 decades, with the identification of oncogenic subgroup of patients who could benefit from targeted therapies. Predominant alterations in NSCLC occur in epidermal growth factor receptor (*EGFR*), mainly located in exon 18 to 21 (Shigematsu and Gazdar, 2006), resulting in tumor cell proliferation, differentiation and migration (Sabbah et al., 2020). The prevalence of all *EGFR* mutations was observed 49.1% in the Asian compared to 11.9%–33.0% in other continents. (Melosky et al., 2022). A classic or common *EGFR* alterations mainly comprise an in-frame deletion in exon 19 and L858R missense mutation in exon 21, accounting for about 80%–90% of *EGFR* alterations in NSCLC (Attili et al., 2022). Approximately 10% of patients with NSCLC harbor uncommon *EGFR* alterations including major uncommon mutation G719X, L861Q and S768I (Figure 1), *de novo* T790M mutation and exon 20 insertions, or their compound forms including the co-existence of common or uncommon mutation (Russo et al., 2019; Zhang et al., 2019; An et al., 2020; Gristina et al., 2020). NSCLC patients with uncommon *EGFR* alterations confer response heterogeneity to diverse *EGFR*-tyrosine kinase inhibitors (TKIs), with a generally poor response to first-generation (1G) *EGFR*-TKIs, but with a favorable response to second-generation (2G) or third-generation (3G) TKIs (Chiu et al., 2015; Xu et al., 2016; Chen et al., 2017; Cho et al., 2020).

Diverse kinds of *EGFR-*TKIs have been approved as the first-line standard therapy for advanced NSCLC with *EGFR-*sensitive mutations in China, including 1G reversible inhibitors erlotinib, gefitinib and icotinib, 2G irreversible and covalent inhibitors afatinib and dacomitinib, and 3G irreversible ones including osimertinib, almonertinib and furmonertinib. According to the molecular structure and biochemical differences among them, sensitivities of uncommon *EGFR* alterations to different *EGFR*-TKIs vary heterogeneously (Beau-Faller et al., 2014; Watanabe et al., 2014; Chiu et al., 2015; Xu et al., 2016; Tu et al., 2017; Zhang et al., 2017; Cho et al., 2020). A series of clinical studies have reported objective response rates (ORRs) of 7%–53.3% to 1G *EGFR*-TKIs for uncommon *EGFR* alterations, with median progression-free survival (mPFS) of 3.0–11.6 months and median overall survival (mOS) of 19.8–25.2 months (Beau-Faller et al., 2014; Watanabe et al., 2014; Xu et al., 2016; Tu et al., 2017; Zhang et al., 2017). Limited studies have issued that they are more responsive to 2G inhibitor afatinib, with ORR of 77.8%, 56%, and 100%, and mPFS of 13.8, 8.2, 14.7 months against G719X, L861Q, and S768I mutations, respectively (Chiu et al., 2015). A recent single-arm prospective phase II study (KCSG-LU15-09) reported that 3G inhibitor osimertinib conferring favorable activity with manageable toxicity in patients with uncommon *EGFR* alterations with an ORR of 53% and mPFS of 8.2 months (Cho et al., 2020). In addition, clinical trials investigating activity of dacomitinib (NCT04504071) and almonertinib (NCT04785742) against uncommon *EGFR* alterations are ongoing, and no prospective data of furmonertinib is available.

Due to the heterogeneity and low incidence of uncommon *EGFR* alterations, as well as the lack of large-scale randomized clinical trials, clinical outcomes of diverse treatment modalities for advanced NSCLC harboring uncommon *EGFR* alterations have not been fully elucidated. Further study is required to evaluate which treatment modality is the most effective for uncommon *EGFR* alterations and which TKI is suitable for the major uncommon *EGFR* alterations to guide precision therapy.

Therefore, we initiated a real-world study to investigate the distribution and therapeutic responses in advanced NSCLC patients harboring uncommon *EGFR* alterations who were treated under four different treatment patterns: 1G *EGFR*-TKIs (gefitinib, erlotinib or icotinib), 2G *EGFR*-TKI afatinib, chemotherapy, and 1G *EGFR*-TKI in combination with chemotherapy. In addition, *in silico* structural models were constructed and molecular dynamics (MD) simulation was performed to predict the sensitivity of major uncommon *EGFR* alteration G719A, S768I, and L861Q to diverse *EGFR*-TKIs.

## Materials and methods

### Patients and data collection

This retrospective study included metastatic or recurrent NSCLC patients harboring *EGFR* uncommon alterations who received treatment pattern including 1G- or 2G- *EGFR*-TKIs, chemotherapy, or 1G *EGFR*-TKIs in combination with chemotherapy as first-line therapy between May 2014 and June 2021 recorded by medical database in Chinese Academy of Medical Sciences (CAMS)/Cancer Hospital. Clinical characteristics, *EGFR* uncommon alteration subtypes, and treatment outcomes from electronic medical records were collected. Testing for *EGFR* alteration was confirmed by the amplification refractory mutation system-polymerase chain reaction (ARMS-PCR) or next-generation sequencing (NGS) based on specimens of tumor tissue or plasma. As an observational study, it was exempted from obtaining patients’ informed consent and was approved by the institutional Ethics Review Board of National Cancer Center/Cancer Hospital, Chinese Academy of Medical Sciences and Peking Union Medical College (approval 18-070/1648).

Eligible patients who met the following criteria were included in the final analysis: age ≥18 years, Eastern Cooperative Oncology Group performance status (ECOG PS) score ≤2, histologically or cytologically confirmed metastatic or recurrent NSCLC with *EGFR* single uncommon or compound uncommon alterations. Compound uncommon alterations were defined as an *EGFR* uncommon missense mutation in combination with another uncommon alteration in exons 18-21. Exclusion criteria included exon 20 insertions or T790M mutation, prior treatment with concurrent chemotherapy and radiotherapy, anti-angiogenic agent combined with *EGFR*-TKIs, or uncontrolled symptomatic brain metastasis.

### Treatment and efficacy evaluation

1G TKIs included gefitinib (a dose of 250 mg once daily), erlotinib (a dose of 150 mg once daily), and icotinib (a dose of 125 mg three times daily), and 2G TKI afatinib was at a dose of 40 mg once daily. The chemotherapy regimens were intravenous pemetrexed (500 mg/m^2^, day 1) plus cisplatin (75 mg/m^2^, d1), with or without anti-vascular endothelial growth factor (*VEGF*) monoclonal antibody (bevacizumab 7.5 mg/kg, day 1) every 21 days as one cycle, followed by maintenance with bevacizumab or pemetrexed monotherapy or their combination after 4-6 cycles. Patients who were intolerable with cisplatin received carboplatin with area under the curve (AUC) equal to 5. Similarly, 1G *EGFR*-TKIs combined with chemotherapy was every 21 days as one cycle, followed by maintenance with pemetrexed and 1G *EGFR*-TKIs after 4-6 cycles. All patients continued treatment until radiographic progression or intolerable toxicity as determined by their physicians, with dose adjustment allowed during the treatment.

Imaging examination at baseline was documented by computed tomography (CT) of the chest and abdomen, brain magnetic resonance imaging (MRI) and whole-body bone scan. Response was evaluated after the first month of treatment initiation and then scanned approximately by every 2 months, which was evaluated by the investigators as a complete response (CR), partial response (PR), stable disease (SD), or progressive disease (PD) according to the Response Evaluation Criteria in Solid Tumors (RECIST) version 1.1. PFS was calculated from the time of treatment initiation to the date of documented progression or death from any cause. OS was defined from the date of first-line treatment to death from any cause or the last follow-up. ORR was the proportion of patients with confirmed CR or PR, and the disease control rate (DCR) was defined as the percentage of those with CR, PR, or SD.

### Structural analysis and molecular dynamics simulation

The homology models of three *EGFR* major uncommon alterations G719A, S768I, and L861Q were computationally constructed based on the crystal structure of human *EGFR* kinase domain by the Schrödinger software (2021-1 Release, Schrödinger Inc., Portland, Oregon) (PDB ID: 4ZAU, 4G5J). The protein was prepared using Maestro software (Schrödinger 2021-1 Release) in the Schrödinger modeling package. Compounds were constructed using the 3D-sketcher module in Maestro. Binding free energy (ΔG_*bind*_) was used to evaluate the binding activity of a certain compound, which was calculated by the GlideScore and molecular mechanics/Generalized Born solvent accessible surface area (MM/GBSA) methods. The detailed calculation regarding ΔG_*bind*_ of a protein-ligand complex was listed in the Appendix (Supplementary Table).

### Statistical analysis

Statistical analyses were carried out by SPSS version 16.0 (SPSS Inc., Chicago, IL, United States) and GraphPad prism software version 5.0 (GraphPad Software Inc., San Diego, CA, United States). Baseline characteristics are presented as descriptive statistics. The Kaplan–Meier method with the long-rank test was performed to compare PFS in different subgroups, which was expressed as the median value and the corresponding 95% confidence index (CI). Univariate and multivariate cox proportional hazards regression were used to evaluate predictive factors associated with PFS. A two-tailed test with *p* < 0.05 was considered statistically significant. Variables included age, sex, smoking history, clinical stage, ECOG PS, histological type, molecular subtype and treatment pattern.

## Results

### Baseline characteristics

A total of 102 eligible patients were included in the final analysis. The baseline characteristics of patients were summarized in Table 1. Sixty-four (62.7%) females were included, and the median age at diagnosis was 60 years old (range: 28–82 years). Ninety-six patients (94.1%) had an ECOG PS of 0 or 1, and 99 (97.1%) patients with lung adenocarcinoma were identified. Most patients had no smoking history (*n* = 71, 69.6%). Nearly a quarter (*n* = 24) presented the central nervous system (CNS) metastasis before initial treatment. Seventy-eight (76.5%) patients were tested for *EGFR* alteration by NGS and others (23.5%) were identified by ARMS-PCR assay. All specimens were available for genetic testing *via* tumor biopsy tissue (*n* = 95) or plasma samples (*n* = 7).

**TABLE 1:** Clinicopathological characteristics of NSCLC patients with *EGFR* uncommon alterations.

Characteristics	Number (%)
Age	
≥65	33 (32.4)
˂65	69 (67.6)
Sex	
Female	64 (62.7)
Male	38 (37.3)
Smoking history	
Current/Former	31 (30.4)
Never	71 (69.6)
Histology	
Adenocarcinoma	99 (97.1)
Non-adenocarcinoma	3 (2.9)
Stage	
Recurrence	9 (8.8)
IV	93 (91.2)
ECOG PS	
0-1	96 (94.1)
2	6 (5.9)
Brain metastases	
Present	24 (23.5)
Absence	78 (76.5)
EGFR testing	
PCR	24 (23.5)
NGS	78 (76.5)
Specimen	
Lung and pleural effusion	81 (79.4)
Lymph nodes	8 (7.8)
plasma	7 (6.9)
Others^a^	6 (5.9)
Treatment pattern	
CT	26 (25.5)
1G-TKI	27 (26.5)
1G-TKI + CT	12 (11.7)
2G-TKI	37 (36.3)

EGFR, epidermal growth factor receptor; NSCLC, non-small cell lung cancer; ECOG PS, Eastern cooperative oncology group performance status; PCR, polymerase chain reaction; NGS, next-generation sequencing; CT, chemotherapy; 1G, first-generation; 2G, second-generation; TKI, tyrosine kinase inhibitors.

a Tumor tissue from brain (3 patients), liver (2 patients) and bone (1 patient).

### Subtypes of epidermal growth factor receptor uncommon alterations

*EGFR* uncommon alterations included single uncommon mutation (e.g., exon 18 p. G719X, exon 20 p. S768I, and exon 21 p. L861Q), compound mutation only comprised of double uncommon mutations (e.g., p. G719X plus p. S768I or other uncommon mutations). The majority of patients presented uncommon plus uncommon mutations (54.9%), while the others were single uncommon mutation (45.1%). Among them, G719X was likely to be combined with additional uncommon alteration. G719X plus S768I was predominantly observed as compound alterations (*n* = 33, 58.9%), followed by E709X (*n* = 10, 17.9%), L861Q/R (*n* = 5, 8.9%), and other uncommon ones (*n* = 8, 14.3%). In addition, single uncommon alterations mainly included G719X (*n* = 36, 78.3%), L861Q (*n* = 5, 10.9%), E709_T710delinsD (*n* = 3, 6.5%), and S768I (*n* = 2, 4.3%). G719X substitutions included G719A, G719C, G719S and other not available subtypes. The detailed molecular subtypes of uncommon *EGFR* alterations were summarized in Table 2.

**TABLE 2:** Molecular subtypes of *EGFR* uncommon alterations.

Uncommon *EGFR* alteration	Number (*N* = 102, %)
Single uncommon alteration	46 (45.1)
G719A/C/S/X	16/2/4/14
L861Q	5
E709_T710delinsD	3
S768I	2
Compound uncommon alteration	56 (54.9)
G719A/C/S/X + S768I	5/12/6/10
G719X + E709 A/K/Q	4/5/1
G719X + L861R/Q	4/1
G719X + R776H	2
G719X + K714 N/E	1/1
G719X + G779C	1
G719A + S720F	1
G719A + L833V	1
L861Q + V769L	1
Total	102 (100)

EGFR, epidermal growth factor receptor.

### Efficacy, safety and survival analysis

At the time of the cutoff date (1 January 2022), the median follow-up time was 38.5 months (range, 1.5–68.6). 1G *EGFR*-TKIs were administered in 27 patients, another 37 patients received afatinib, 26 patients received chemotherapy, and 12 patients received a 1G *EGFR*-TKIs combined with chemotherapy. The ORR to 1G *EGFR*-TKIs, afatinib, chemotherapy, 1G *EGFR*-TKIs combined with chemotherapy was 29.6%, 59.5%, 30.8%, and 50.0% (*p* = 0.049), and DCR was 81.5%, 81.1%, 57.6%, and 83.3%, respectively (*p* = 0.112).

In the afatinib group, any grade of treatment-related side effects (TRAEs) included diarrhea (*n* = 27, 73.0%), rash (*n* = 25, 67.6%), oral mucosal toxicity (*n* = 16, 43.2%), nausea and vomiting (*n* = 2, 5.4%). The majority of TRAEs were grade 1 or 2, and grade 3 TRAEs were diarrhea (*n* = 1, 2.7%) and rash (*n* = 3, 8.1%). No grade 4 or 5 TRAEs were reported. One patient (2.7%) discontinued afatinib because of intolerable diarrhea. Dose reductions from 40 to 30 mg were observed in 10 patients and reduction to 15 mg among 2 patients. For 1G EGFR-TKIs with chemotherapy, neutropenia, anemia, and thrombocytopenia were more common, the rates of grade 3 for these hematological toxicities were 33.3% (*n* = 2), 25.0% (*n* = 3), and 16.7% (*n* = 2). Meanwhile, liver dysfunction was observed 16.7% (*n* = 2).

**FIGURE 2:**
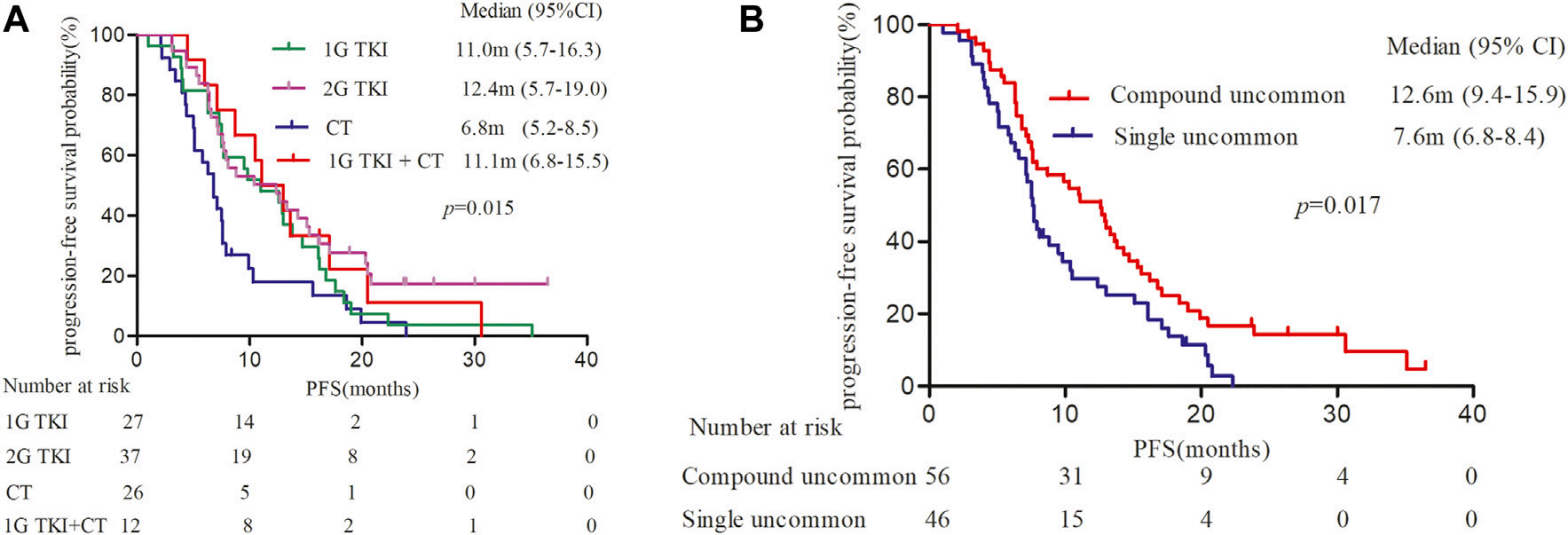
Kaplan-Meier curves of progression-free survival (PFS) in patients harboring EGFR uncommon alterations. **(A)** treated with different treatment modalities. **(B)** with different uncommon EGFR subtypes.

It was observed mPFS differed significantly between different treatment patterns (*p* = 0.015). The mPFS of 1G *EGFR*-TKIs, afatinib, chemotherapy, and 1G *EGFR*-TKIs in combination with chemotherapy were 11.0 (95% CI, 5.7–16.3), 12.4 (95% CI, 5.7–19.0), 6.8 (95% CI, 5.2–8.5), and 11.1 month (95% CI, 6.8–15.5), respectively (Figure 2A). We further analyzed clinical outcomes in advanced NSCLC patients harboring uncommon *EGFR* subtypes. Intriguingly, patients harboring compound *EGFR* uncommon mutations achieved significantly longer mPFS compared with single *EGFR* uncommon mutations (12.6 months, 95% CI: 9.4–15.9 months vs. 7.6 months, 95%CI: 6.8–8.4 months, *p* = 0.017) (Figure 2B). The median OS has not reached in any subgroup.

### Univariate and multivariate analyses for progression-free survival

Univariate analysis showed that the PFS was significantly associated with molecular subtypes (*p* = 0.018), treatment modalities (*p* = 0.019), and ECOG PS (*p* = 0.006) (Table 3). Multivariate analyses verified that *EGFR* molecular subtypes, treatment patterns and ECOG PS were independent predictors of PFS for advanced NSCLC patients with *EGFR* uncommon alterations (*p* < 0.05, Table 4).

**TABLE 3:** Univariate survival analyses for PFS.

Variable	B	SE	HR	95% CI	P
Age (≥65 vs. <65)	−0.145	0.228	0.865	0.553–1.353	0.525
Sex (Male vs. Female)	−0.087	0.216	0.916	0.600–1.400	0.686
Smoking (Current/Former vs. Never)	−0.197	0.229	0.821	0.524–1.286	0.388
ECOG PS (0-1 vs. 2)	−1.195	0.438	0.303	0.128–0.714	0.006
Baseline brain metastases	−0.212	0.247	0.809	0.498–1.312	0.389
(Present vs. Absence)					
Molecular subtype					
(Compound uncommon vs. Single uncommon)	−0.507	0.214	0.603	0.396–0.917	0.018
Treatment pattern					0.019
(CT vs. 2G TKI)	0.848	0.277	2.334	1.357–4.016	0.0020.
(1G TKI vs. 2G TKI)	0.362	0.269	1.437	0.848–2.435	0.178
(1G TKI + CT vs. 2G TKI)	0.121	0.356	1.129	0.562–2.265	0.734

HR, hazard ratio; CI, confidence interval; ECOG PS, Eastern Cooperative Oncology Group performance status; TKI, tyrosine kinase inhibitor; CT, chemotherapy; 1G, first-generation; 2G, second-generation.

**TABLE 4:** Predictors for PFS by multivariate Cox regression.

Variable	B	SE	HR	95% CI	*P*
ECOG PS (0-1 vs. 2)	−1.546	0.452	0.213	0.088–0.517	0.001
Molecular subtype					
(Compound uncommon vs. Single uncommon)	−0.653	0.222	0.521	0.337–0.805	0.003
Treatment pattern					0.002
(CT vs. 2G TKI)	1.110	0.288	3.034	1.726–5.334	0.000
(1G TKI vs. 2G TKI)	0.583	0.278	1.791	1.039–3.086	0.036
(1G TKI + CT vs. 2G TKI)	0.344	0.368	1.410	0.686–2.898	0.350

HR, hazard ratio; CI, confidence interval; ECOG PS, Eastern Cooperative Oncology Group performance status; TKI, tyrosine kinase inhibitor; CT, chemotherapy; 1G, first-generation; 2G, second-generation.

### Computational models and tyrosine kinase inhibitors binding activity

**FIGURE 3:**
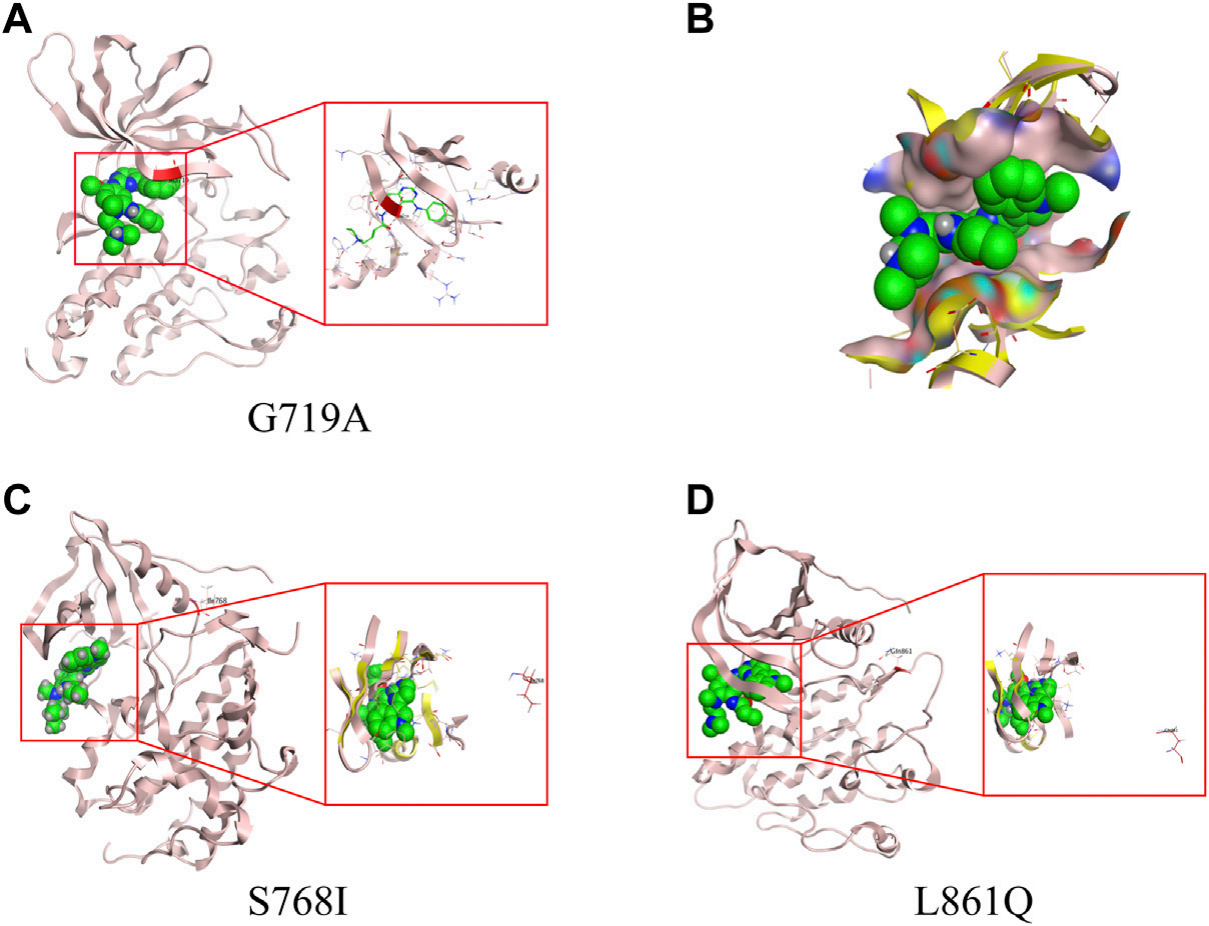
3D-based homology models with different uncommon *EGFR* subtypes. **(A)** G719A conformation. **(B)** drug-binding pocket of G719A (pink) and EGFR wild type (yellow). Protein structure and drug-binding pocket of conformation S768I **(C)** and L861Q **(D)**.

From homology model of G719A, Ala719 (labelled in red) is located in the binding pocket (Figure 3A), which has a direct effect on the binding of TKI and a slight change of amino acids might induce a different drug-binding activity. Compared with the wild type (WT) *EGFR* kinase, both glycine and alanine are relative small-molecule amino acids, and there is no obvious steric hindrance caused by G719A conformation. Distance between the mutant protein Ala719 (pink) and the small molecule (green) is closer than that observed in the WT Gly719 (yellow), which indicates a narrower binding pocket, and might affect the binding of the small molecule (Figure 3B). By comparison, Ile768 is far away from the binding pocket, and S768I conformation could not affect the binding of small molecules, owing to its location different with WT *EGFR* kinase (Figure 3C). Similarly, Gln861 is also far away from the binding pocket when compared with WT *EGFR* kinase (Figure 3D), indicating an unaffected binding activity induced by L861Q conformation.

**FIGURE 4:**
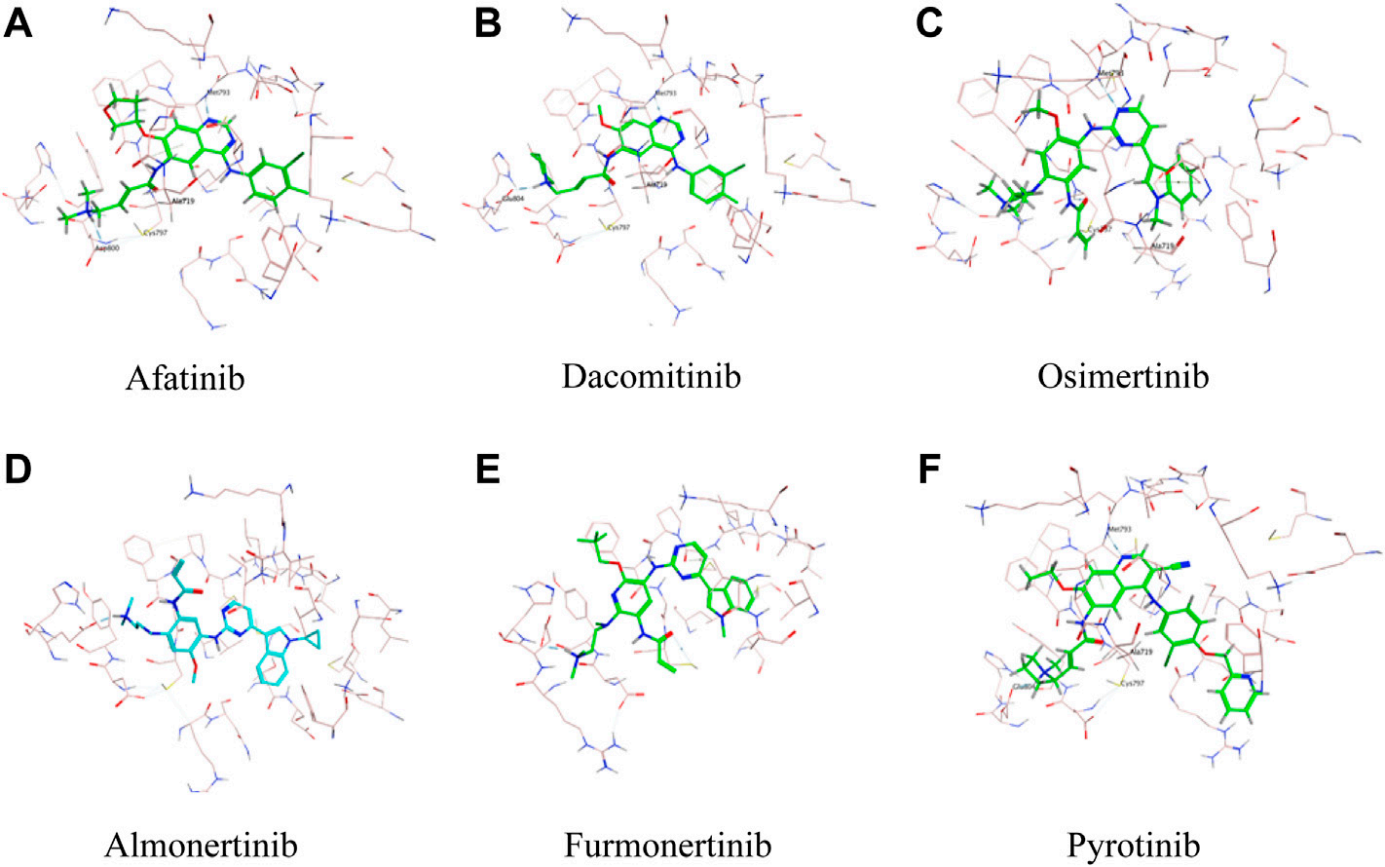
Binding modes of different TKIs Afatinib **(A)**, Dacomitinib **(B)**, Osimertinib **(C)**, Almonertinib **(D)**, Furmonertinib **(E)**, and Pyrotinib **(F)** to G719A conformation.

As was illustrated in Table 5, afatinib (Figure 4A), dacomitinib (Figure 4B) and osimertinib (Figure 4C) revealed favorable binding activity to G719A conformation *via* H-bond in Met793, covalent bond in Cys797, salt-bridge, and hydrophobic interactions. Notably, another two 3G *EGFR-*TKI almonertinib (Figure 4D) and furmonertinib failed to bind G719A (Figure 4E), which might be attributed to the lackness of molecular interaction. Pyrotinib, a pan-*ErbB* inhibitor which was designed to target *HER2* in breast cancer and NSCLC, showed potent binding activity to G719A (Figure 4F), which was observed conferring similar molecular interactions like afatinib, dacomitinib and osimertinib. For another two uncommon alteration S768I and L861Q, both these 2G or 3G *EGFR-*TKIs and pyrotinib demonstrated favorable binding activities (Figure. S1, S2).

**TABLE 5:** Binding free energies (ΔGbind, kcal/mol) of different TKIs for EGFR major uncommon mutations by dynamics calculation.

Molecule	G719A		S768I		L861Q	
	GlideScore	MM/GBSA	GlideScore	MM/GBSA	GlideScore	MM/GBSA
Gefitinib	−5.7	−77.3	−7.4	−79.2	−6.3	−83.4
Erlotinib	−6.5	−82.7	−7.9	−85.5	−7.4	−89.2
Icotinib	−6.0	−73.4	−7.2	−75.7	−6.4	−80.6
Afatinib	−6.8	−89.1	−7.8	−87.1	−8.9	−95.1
Dacomitinib	−7.0	−81.0	−7.2	−77.3	−8.7	−92.2
Osimertinib	−6.6	−80.2	−8.0	−87.3	−8.9	−89.3
Almonertinib	−5.9	−75.6	−8.0	−89.1	−8.5	−87.3
Furmonertinib	−6.2	−79.9	−8.2	−86.4	−8.4	−77.6
Pyrotinib	−6.7	−90.9	−6.7	−80.9	−7.5	−73.7

## Discussion

In this study, we analyzed the distribution of *EGFR* uncommon subtypes and clinical outcomes of different treatment strategies as first-line therapy. Notably, we provided molecular structures and gave insights into binding activities of G719A, S768I, and L861Q mutations to different *EGFR*-TKIs. This study demonstrated that the majority of uncommon *EGFR* alterations were compound mutations in the form of double uncommon forms (54.9%). G719X plus S768I was the most common partner observed in compound uncommon alterations. Similar to the previous study, G719X/S768I was the most frequent subtype in double uncommon mutations, and it seemed that single *EGFR* uncommon mutation had a higher probability to form a compound alteration (Si et al., 2021).

In addition, multivariate analyses in this study demonstrated that treatment patterns, *EGFR* mutation subtypes, and ECOG PS were independent predictors of PFS in NSCLC patients with uncommon *EGFR* alterations. The mPFS treated with different treatment strategies was significantly different (*p* = 0.015). A significant deficit in PFS was observed in patients who received chemotherapy monotherapy (mPFS, 6.8 months). The mPFS of patients treated with afatinib was 12.4 months. There was no significant difference in PFS between 1G TKIs combined with chemotherapy and afatinib subgroups (*p* > 0.05). In an expansion cohort study evaluating responses of *EGFR* exon 18 mutations to diverse treatment strategies, afatinib and 1G TKIs in combination with chemotherapy prolonged PFS in patients who harbored uncommon *EGFR* alterations (Xu et al., 2021). To some degree, each treatment modality could be an optional strategy. An *in vitro* study as well showed a better response to afatinib in cells with G719X, S768I and L861Q (Banno et al., 2016; Kobayashi and Mitsudomi, 2016). Similarly, the most comprehensive report to date also demonstrated that single and compound uncommon alterations both could benefit from afatinib (Yang et al.,.2020). In addition, several real-word studies have further verified the activity of afatinib against *EGFR* uncommon alterations (Li et al., 2021; Hang et al., 2022).

Intriguingly, molecular subtype was associated with PFS in our cohort study. Previous study reported that gefitinib had quite variable growth-suppressive effects on different *EGFR*-expressing cells, and the kinase domain of *EGFR* subtypes may alter drug responsiveness in NSCLC (Chen et al., 2006). Our study is in line with previous observations that patients harboring single uncommon mutation had a median PFS of 7.6 months, while those with double uncommon alterations had a relatively longer PFS of 12.6 months, and significant statistical difference was observed (*p* = 0.017). Therefore, NGS testing is highly recommended so as to predict heterogeneous responses to *EGFR* TKIs for each specific *EGFR* uncommon subtype.

Based on the LUX-Lung clinical trials, afatinib is currently approved for the treatment of metastatic NSCLC harboring *EGFR* S768I, L861Q and/or G719X alterations by the U.S. Food and Drug Administration (FDA) in 2018. In addition to afatinib, another 2G covalent *EGFR* inhibitor dacomitinib, and 3G *EGFR-*TKI osimertinib, almonertinib, and furmonertinib are widely used in China. In our study, the computational models and TKI binding activity revealed that a slight change of amino acids could induce a different drug-binding activity. Compared with the WT of *EGFR*, G719A mutation may affect the binding of small molecular TKIs due to the narrow binding pocket. 2G-TKIs afatinib, dacomitinib, 3G-TKI osimertinib and pan-*ErbB* inhibitor pyrotinib both revealed favorable binding activity to G719A mutation. Notably, another two 3G-TKIs almonertinib and furmonertinib failed to bind G719A due to the lackness of molecular interaction. Both these 2G- and 3G*-*TKIs and pyrotinib demonstrated favorable binding activities to S768I and L861Q. An *in vitro* sensitivities of Ba/F3 cells also showed a better response to afatinib and osimertinib than to gefitinib and erlotinib with G719X, S768I and L861Q (Kobayashi et al., 2016). Another pan-*ErbB* inhibitor neratinib was observed promising activity with overall ORR of 60% and mPFS of 9.1 months among NSCLC patients with *EGFR* exon 18 mutations, suggesting a potential role for neratinib as a systemic treatment option for patients with NSCLC and difficult-to-treat uncommon mutations (Goldman et al., 2021). A series of studies showed that dacomitinib, as well as afatinib, was active against G719X mutation and had a remarkable efficacy in later-line treatment in NSCLC patients (Kobayashi et al., 2015; Morita et al., 2021). Consistent with the previous reports, a retrospective study and case studies also suggested that these patients could be response to osimertinib in real-word study (Ji et al., 2020; Okuno et al., 2020). We speculate that afatinib, dacomitinib, and osimertinib might confer favorable activity to G719A, S768I, and L861Q, whereas almonertinib and furmonertinib revealed less activity to G719A. Based on the computational modeling, the analysis for binding activity of *EGFR* uncommon alterations are used to predict the efficacy as a powerful tool in precision medicine to help selecting effective drugs for these patients.

A number of limitations must be noted. Firstly, this was a retrospective study with a limited sample size, which might have induced potential bias. In addition, 1G *EGFR*-TKIs involved gefitinib, erlotinib and icotinib, and *EGFR* alterations was confirmed by different detection methods, including ARMS-PCR or NGS based on specimens of tumor tissue or plasma samples, and selection bias was inevitable. Given the limited sample size, we did not analyze the efficacy of another 2G *EGFR*-TKI dacomitinib, or the 3G *EGFR*-TKIs osimertinib, almonertinib, and furmonertinib. Furthermore, the analysis for binding activity of *EGFR* uncommon alterations based on computational modeling would become a powerful tool in precision medicine to help selecting effective drugs for these patients, but it cannot replace clinical evidence and still requires further real-world clinical data confirmations.

In conclusion, this study indicated that the combination of 1G *EGFR*-TKIs with chemotherapy or afatinib monotherapy was associated with a favorable response and promising PFS benefit for NSCLC patients with uncommon *EGFR* alterations. G719A mutation might induce a different drug-binding activity, while S768I and L861Q alterations indicated an unaffected TKI binding activity. Therefore, different 3G-TKIs may have their different therapeutic efficacy. Further studies should be performed to determine the most appropriate treatment recommendation for NSCLC patients harboring uncommon *EGFR* alterations.

## Data Availability

The original contributions presented in the study are included in the article and Supplementary Material, and further inquiries can be directed to the corresponding author.

## Supplementary material

SUPPLEMENTARY FIGURE S1 Binding modes of different TKIs Afatinib **(A)**, Dacomitinib **(B)**, Osimertinib **(C)**, Almonertinib **(D)**, Furmonertinib **(E)**, and Pyrotinib **(F)** to S768I conformation.

SUPPLEMENTARY FIGURE S2 Binding modes of different TKIs Afatinib **(A)**, Dacomitinib **(B)**, Osimertinib **(C)**, Almonertinib **(D)**, Furmonertinib **(E)**, and Pyrotinib **(F)** to L861Q conformation.
