# Supplementary figures and images for: EGFR uncommon alterations in advanced non-small cell lung cancer and structural insights into sensitivity to diverse tyrosine kinase inhibitors
Source: Front Pharmacol. 2022 Sep 16;13:976731. doi: 10.3389/fphar.2022.976731 (PMC9523264; doi:10.3389/fphar.2022.976731)

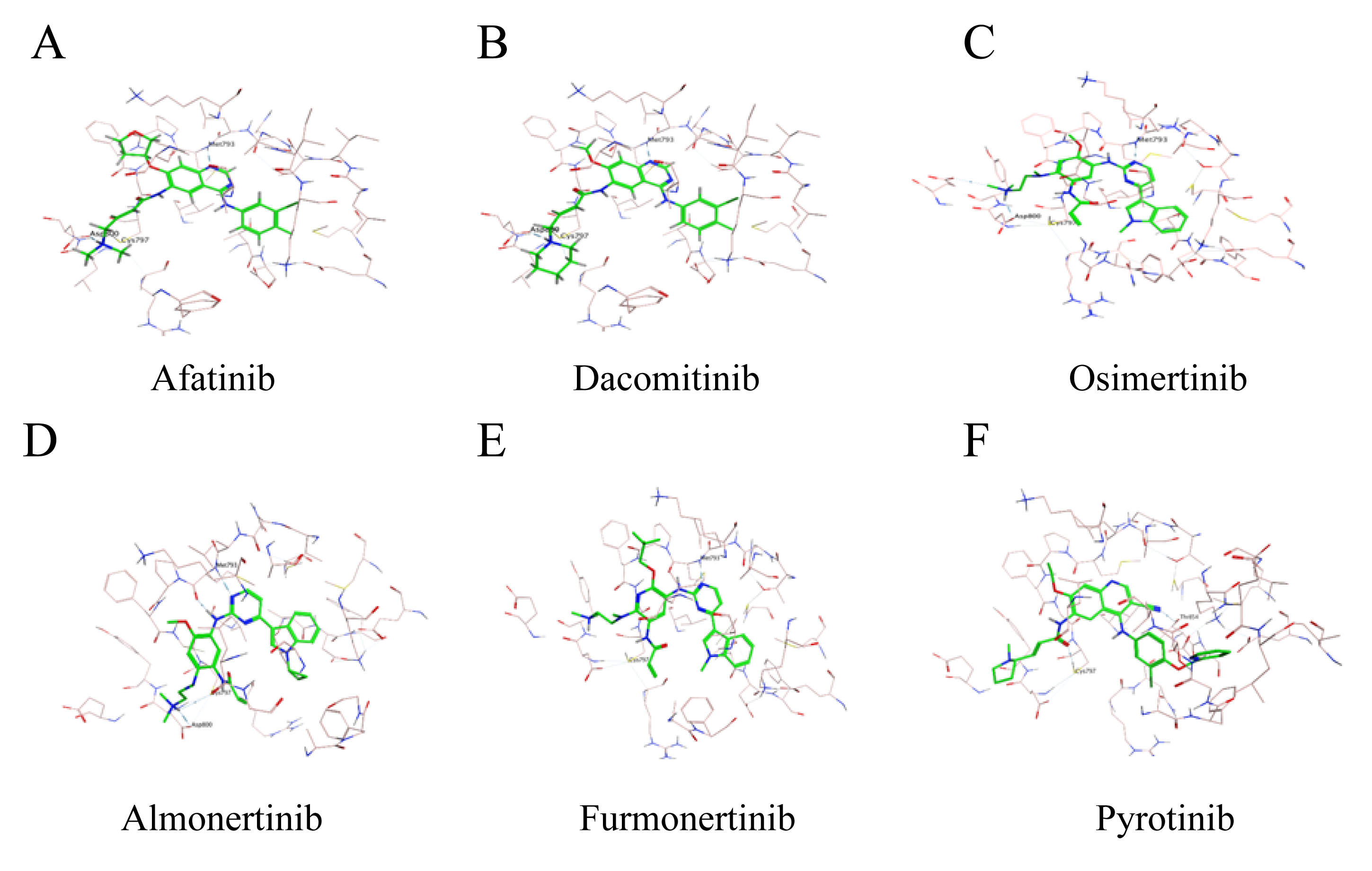

Supplement: Supplementary file 1 [file Image2.TIF]

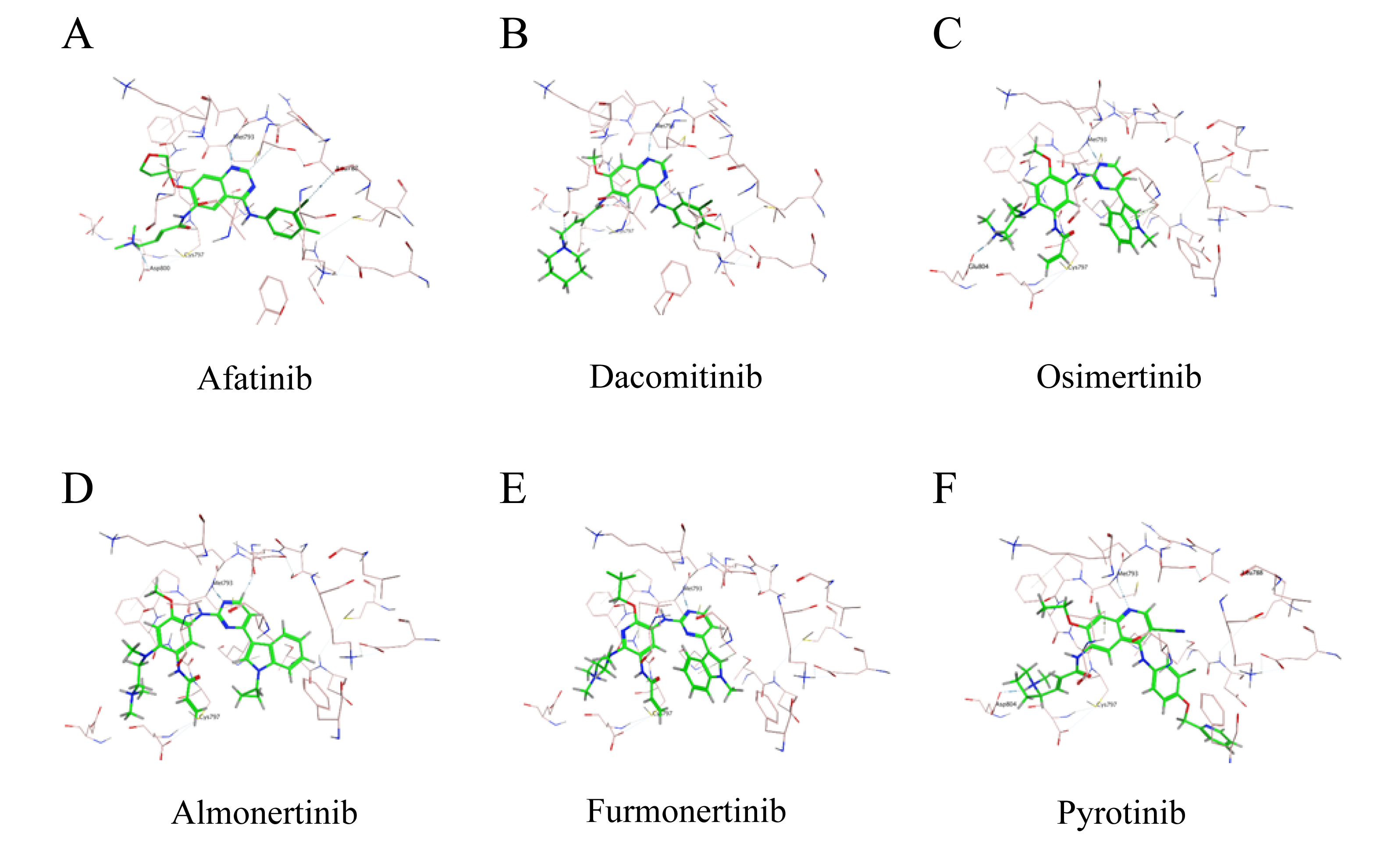

Supplement: Supplementary file 2 [file Image1.TIF]
